# Correction methods for noncontact intraocular pressure measurement in patients with keratoconus and healthy individuals

**DOI:** 10.5935/0004-2749.20220072

**Published:** 2025-08-22

**Authors:** Azam Alvani, Hassan Hashemi, Mohammad Pakravan, Mehdi Yaseri, Ebrahim Jafarzadehpur, Akbar Fotouhi

**Affiliations:** 1 Noor Ophthalmology Research Center, Noor Eye Hospital, Tehran, Iran; 2 Department of Ophthalmology, School of Medicine, Tehran University of Medical Sciences, Tehran, Iran; 3 Ophthalmic Epidemiology Research Center, Shahid Beheshti University of Medical Sciences, Tehran, Iran; 4 Department of Epidemiology and Biostatistics, School of Public Health, Tehran University of Medical Sciences, Tehran, Iran; 5 Department of Optometry, School of Rehabilitation, Iran University of Medical Sciences, Tehran, Iran

**Keywords:** Intraocular pressure, Noncontact tonometry, Cornea, Corneal pachymetry, Keratoconus, Pressão intraocular, Tonometria sem contato, Córnea, Paquimetria corneana, Ceratocone

## Abstract

**Purpose:**

The objective of this study was to investigate the usefulness of four different algorithms to correct noncontact intraocular pressure measurement errors in keratoconus patients and normal individuals.

**Methods:**

Noncorrected intraocular pressure and corrected intraocular pressures were measured in one eye of 34 patients with keratoconus and 34 ageand gender-matched healthy controls using Corvis Scheimpflug Technology. The correlation of noncorrected intraocular pressure and corrected intraocular pressures with age, axial length, corneal shape, thickness, and biomechanics was calculated. Corrected intraocular pressures were compared with noncorrected intraocular pressure using paired *t* test and Bland-Altman plots (95% limits of agreement).

**Results:**

The noncorrected intraocular pressure correlated with corneal thickness and biomechanical parameters in both groups (all *p*≤0.047), and front and back mean keratometry in the keratoconus group (r=-0.39, p=0.02, and r=0.39, p=0.02, respectively). After adjustment with different intraocular pressure correction algorithms, biomechanically corrected intraocular pressure showed a minimal correlation with corneal features and a nonsignificant difference with noncorrected intraocular pressure in the healthy group (-0.1 ± 1.1 mmHg, p=0.58; 95% limits of agreement: -2.3 to 2.1 mmHg).

**Conclusions:**

Measuring intraocular pressure using noncontact tonometry and its corrected forms with a corneal thickness-based simple linear formula in patients with keratoconus is associated with many errors. Using more complex formulas that take into consideration more corneal stiffness parameters in addition to corneal thickness, such as biomechanically corrected intraocular pressure formula, may be more reliable and beneficial in this group of patients.

## Abstract (pt)

**Objetivo:**

Investigar a utilidade de quatro algoritmos diferentes para corrigir erros de medição sem contato da pressão intraocular em pacientes saudáveis e com ceratocone.

**Métodos:**

A pressão intraocular não corrigida e as pressões intraoculares corrigidas foram medidas em um olho de 34 pacientes com ceratocone e 34 pacientes do grupo controle saudável pareados por idade e gênero usando a tecnologia Corvis Scheimpflug. Foi calculada a correlação da pressão intraocular não corrigida e das pressões intraoculares corrigidas com idade, comprimento axial e formato, espessura e biomecânica da córnea. As pressões intraoculares corrigidas foram comparadas com a pressão intraocular não corrigida usando o teste t pareado, e gráficos de Bland-Altman (limites de concordância de 95%).

**Resultados:**

A pressão intraocular não corrigida correlacionou-se com a espessura da córnea e com os parâmetros biomecânicos em ambos os grupos (todos p≤0,047) e a ceratometria média frontal e posterior no grupo com ceratocone (r=-0,39, p=0,02, r=0,39, p=0,02, respectivamente). Após o ajuste com diferentes algoritmos de correção da pressão intraocular, a pressão intraocular corrigida biomecanicamente revelou uma correlação mínima com as características da córnea e uma diferença não significativa com a pressão intraocular não corrigida no grupo saudável (-0,1 ± 1,1 mmHg, p=0,58; limites de concordância de 95%: -2,3 a 2,1 mmHg).

**Conclusões:**

A medição da pressão intraocular usando tonometria sem contato e suas formas corrigidas usando fórmulas lineares, simples, baseadas na espessura da córnea em pacientes com ceratocone estão associadas a muitos erros. O uso de fórmulas mais complexas que consideram mais parâmetros de rigidez da córnea além da espessura da córnea, como fórmula de pressão intraocular corrigida biomecanicamente, pode ser mais confiável e benéfico neste grupo de pacientes.

## INTRODUCTION

Keratoconus is the most common primary corneal ectasia, with an estimated prevalence of 2.3% in the general population^(1)^. Because of its effect on corneal shape, thickness, and biomechanics, intraocular pressure (IOP) measurement using applanation tonometry devices is associated with significant unpredictable errors in these patients, which may postpone a diagnosis of glaucoma^(2,3)^. Noncontact applanation tonometers such as Corvis Scheimpflug Technology (CST) are very popular and widely used as glaucoma-screening tools. In addition to their quick and easy use, they simultaneously measure the central corneal thickness (CCT) and biomechanics and adjust the measured IOP (noncorrected IOP [IOPnct]) according to predefined formulas^(4,5)^. Therefore, these tonometers might be more beneficial in corneas with abnormally altered characteristics, such as keratoconus corneas. However, the efficacy of IOP correction formulas has not yet been studied in these patients.

It seems that a more accurate estimate of true IOP can be obtained using IOP measurement or correction methods that minimize the dependence of measured IOP on the corneal curvature, thickness, and biomechanics^(6)^. To determine such methods, we conducted this study to compare the IOPnct and corrected IOPs obtained by CST in patients with keratoconus and healthy individuals. We also evaluated the correlation coefficient of IOPnct and corrected IOPs with corneal stiffness parameters.

## METHODS

This case-control study was conducted at Noor Eye Hospital, Tehran, Iran, and was approved by the Ethics Committee of Tehran University of Medical Sciences (E.C. Ref No.: IR.TUMS.VCR.REC.1396.4621). All procedures followed the guidelines of the Declaration of Helsinki. The protocol of the study was explained to the participants, and written informed consent was obtained from all individuals before the study.

The keratoconus group included patients referred to the keratoconus unit. Ageand gender-matched healthy controls were selected from among hospital staff members who volunteered to participate in the study. Eligibility criteria in both groups were age between 18 and 45 years; no history of ocular surgery, trauma, corneal scars, or pathologies; and no history of systemic autoimmune diseases or diabetes. For all participants, we performed full optometry and ophthalmology examinations, including visual acuity testing, refraction, slit-lamp biomicroscopy, and fundoscopy, and one eye of each participant was recruited in the study. In the keratoconus group, if both eyes met the eligibility criteria, one eye was randomly selected using a computer-generated table of random numbers. In the healthy group, the right or left eye was selected in such a way that it matched the keratoconus group. Additional examinations including noncontact ocular biometry (IOLMaster 500, Carl Zeiss, Jena, Germany), computerized tomography (Pentacam, Oculus Optikgerate GmbH, Wetzlar, Germany), and CST noncontact tonometry (Corvis Scheimpflug Technology, software version 1.4r1755, Oculus Optikgerate GmbH, Wetzlar, Germany) were performed for the recruited eye.

To minimize the confounding effects of diurnal variations in IOP and corneal thickness, we performed all measurements between 10:00 AM and 2:00 PM, at least 2 hours after the patient’s wake-up time^(7)^.

The CST measures IOP based on information regarding corneal deformation in response to a precisely aligned and parallelized jet of air. Then, the measured IOP (IOPnct) is corrected using the CCT-based IOP correction formulas, including Ehlers^(8)^, Shah^(9)^, and Dresden^(10)^. In addition, a biomechanically corrected IOP (bIOP) is also provided using the deformation data, CCT, and age^(5)^. We performed the examination with CST with the patient in a sitting position and without anesthesia. The quality of the measurements was proven by the appearance of the “OK” quality index on the device screen.

We performed all statistical analyses using SPSS software (version 23.0, IBM, Chicago, IL, USA). We used the Kolmogorov-Smirnov test to evaluate the distribution of the variables. Independent *t* test or Mann-Whitney *U* test was used to compare the mean values between the keratoconus and normal groups. We compared the IOPnct and corrected IOPs in each of the keratoconus and normal groups using the paired *t* test.

The agreement between the IOPnct and corrected IOPs in each group was evaluated using the Bland-Altman plots, with 95% limits of agreement. Pearson’s correlation coefficient was used to examine the correlation of IOPnct and corrected IOPs with age, axial length (AL), and corneal features. A p-value less than 5% was considered statistically significant.

## RESULTS

We enrolled a total of 34 eyes of 34 keratoconus patients and 34 eyes of 34 normal controls. The mean participant age was 32 ± 5 years in the keratoconus group and 32 ± 4 years in the healthy group, with no significant difference between the groups (p=0.66). In both groups, 74% of the participants were men. The mean refractive error (spherical equivalent) and mean AL were -3.38 ± 2.89 diopter (D) and 24.08 ± 1.14 mm in the keratoconus group, which were significantly higher as compared with 0.02 ± 0.84 D and 23.33 ± 0.85 mm in the healthy group (p<0.001 and p=0.003, respectively).

Table 1 presents the data on corneal shape, thickness, and biomechanics as well as IOP measurements in the keratoconus and healthy groups. In terms of corneal shape and thickness, patients with keratoconus had a higher maximum keratometry (K_max_), higher front and back mean keratometry (K_m_), and lower CCT (all p<0.001). In terms of corneal biomechanical parameters, patients with keratoconus had a higher deformation amplitude (DA) and peak distance (PD) and a lower radius of curvature (RC) (all p≤0.03). With regard to IOP measurements, the keratoconus group showed a significantly lower IOPnct (p=0.003), although there was no significant difference in corrected IOPs, including IOPEhlers, IOPShah, IOPDresden, and bIOP, between the two groups (all *p*≥0.05).

**Table 1:** Comparison of corneal features and tonometry findings in patient with keratoconus and age-matched control subjects

Parameter			Keratoconus (Mean ± SD)	Healthy control (Mean ± SD)	p value
**Corneal shape**		**K**_max_ **(diopter)**	52.71 ± 5.51	44.82 ± 1.79	<0.001
		**Front K**_m_ **(diopter)**	46.41 ± 3.31	43.69 ± 1.65	<0.001
		**Back K**_m_ **(diopter)**	-6.81 ± 0.71	-6.29 ± 0.26	<0.001
**Corneal thickness**		**CCT (**µ**m)**	480 ± 35	537 ± 35	<0.001
**Corneal biomechanics**		**Deformation amplitude (mm)**	1.06 ± 0.11	0.94 ± 0.12	<0.001
		**Peak distance (mm)**	5.04 ± 0.23	4.89 ± 0.33	0.03
		**Radius of curvature (mm)**	6.88 ± 0.96	8.58 ± 1.02	<0.001
**Intraocular pressure**		**IOPnct (mmHg)**	14.2 ± 2.5	16.4 ± 3.3	0.003
		**IOPEhlers (mmHg)**	18.4 ± 2.9	17 ± 2.8	0.05
		**IOPShah (mmHg)**	17.4 ± 2.5	17 ± 2.7	0.53
		**IOPDresden (mmHg)**	16.7 ± 2.5	17 ± 2.7	0.66
		**bIOP (mmHg)**	15.4 ± 2.4	16.3 ± 2.6	0.13

*K*_max_= maximum keratometry; *Front K*_m_ front mean keratometry; *Back K*_m_= back mean keratometry; *CCT=* central corneal thickness; *IOPnct=* intraocular pressure not corrected; *bIOP=* biomechanically corrected IOP; *SD=* standard deviation.

Table 2 shows the Pearson’s correlation coefficient test results for determining the correlation of IOPnct and corrected IOPs with age, AL, and corneal features in each group. There was no correlation between IOPnct and corrected IOPs with the parameters of age, AL, and K_max_ in the keratoconus and healthy groups (all p≥0.06). IOPnct was negatively correlated with front K_m_ (r=-0.39, p=0.02) and positively correlated with back K_m_ in the keratoconus group (r=0.39, p=0.02). The relationships between other IOP readings and front and back K_m_ were not statistically significant in the two groups (all p≥0.05). IOPnct was positively correlated and IOPEhlers negatively correlated with CCT in both the keratoconus group (r=0.34, p=0.047; and r=-0.46, p=0.009, respectively) and healthy group (r=0.46, p=0.006; and r=-0.37, p=0.03, respectively). Correlations between other IOP readings and CCT were not statistically significant in either group (all p≥0.19). IOPnct and all corrected IOPs had a negative correlation with DA (r=-0.58 to -0.87, all p<0.001) and PD (r=-0.47 to -0.83, all p≤0.008), and all had a positive correlation with RC (r=0.39 to 0.66, all p≤0.02) in both the keratoconus and healthy groups, except for IOPEhlers in the keratoconus group (p=0.08).

**Table 2:** Correlations between noncorrected and corrected intraocular pressure with age, axial length, and corneal features in patients with keratoconus and age-matched controls

Parameter	Group	Age	Axial length	Kmax	Front K_m_	Back K_m_	CCT	Deformation amplitude	Peak distance	Radius of curvature
**IOPnct**	**KC**	-0.14 (0.44)	0.15 (0.39)	-0.32 (0.06)	**-0.39** **(0.02)**	**0.39 (0.02)**	**0.34** **(0.047)**	**-0.87** **(<0.001)**	**-0.69** **(<0.001)**	**0.59** **(<0.001)**
	**Healthy**	-0.08 (0.64)	-0.16 (0.38)	0.21 (0.23)	0.15 (0.41)	-0.22 (0.20)	**0.46** **(0.006)**	**-0.87** **(<0.001)**	**-0.83** **(<0.001)**	**0.66** **(<0.001)**
**IOPEhlers**	**KC**	0.13 (0.49)	0.17 (0.35)	0.07 (0.71)	-0.05 (0.79)	0.10 (0.61)	**-0.46** **(0.009)**	**-0.59** **(<0.001)**	**-0.47** **(0.008)**	0.32 (0.08)
	**Healthy**	-0.08 (0.66)	-0.14 (0.44)	0.33 (0.06)	0.31 (0.08)	-0.26 (0.14)	**-0.37** **(0.03)**	**-0.58** **(<0.001)**	**-0.49** **(0.004)**	**0.39 (0.02)**
**IOPShah**	**KC**	0.06 (0.76)	0.19 (0.30)	-0.05 (0.78)	-0.17 (0.36)	0.20 (0.28)	-0.24 (0.19)	**-0.73** **(<0.001)**	**-0.58** **(0.001)**	**0.43 (0.02)**
	**Healthy**	-0.09 (0.63)	-0.13 (0.47)	0.29 (0.10)	0.25 (0.16)	-0.25 (0.16)	-0.12 (0.51)	**-0.74** **(<0.001)**	**-0.65** **(<0.001)**	**0.52** **(0.002)**
**IOPDresden**	**KC**	-0.02 (0.92)	0.17 (0.34)	-0.10 (0.56)	-0.19 (0.29)	0.24 (0.17)	-0.17 (0.33)	**-0.79** **(<0.001)**	**-0.62** **(<0.001)**	**0.50** **(0.003)**
	**Healthy**	-0.09 (0.62)	-0.19 (0.29)	0.32 (0.06)	0.28 (0.11)	-0.29 (0.10)	0.04 (0.85)	**-0.80** **(<0.001)**	**-0.73** **(<0.001)**	**0.58** **(<0.001)**
**bIOP**	**KC**	-0.12 (0.53)	0.16 (0.37)	-0.21 (0.24)	-0.31 (0.09)	0.35 (0.05)	0.07 (0.69)	**-0.84** **(<0.001)**	**-0.69** **(<0.001)**	**0.57** **(0.001)**
	**Healthy**	-0.13 (0.47)	-0.18 (0.31)	0.28 (0.11)	0.24 (0.17)	-0.27 (0.12)	0.18 (0.32)	**-0.85** **(<0.001)**	**-0.78** **(<0.001)**	**0.62** **(<0.001)**

*K*_max_= maximum keratometry; *Front K*_m_= front mean keratometry; *Back K*_m_= back mean keratometry; *CCT=* central corneal thickness; *IOPnct*= intraocular pressure not corrected; *KC=* keratoconus; *bIOP=* biomechanically corrected IOP.

All data are presented as *r* (*p* value).

Bold values are statistically significant.

Table 3 shows the mean difference between IOPnct and corrected IOPs using different correction formulas and their 95% limits of agreement in the keratoconus and healthy groups. Bland-Altman scatter plots in figures 1 and 2 demonstrate the agreement between IOPnct and different corrected IOPs in the keratoconus and healthy groups. According to the results, the best agreement with IOPnct was observed for bIOP in both the keratoconus and healthy groups (95% limits of agreement: -0.5 to 2.7, and -2.3 to 2.1 mmHg, respectively). However, although the mean difference between IOPnct and bIOP was not significantly different from zero in the healthy group (paired *t* test, -0.1 ± 1.1 mmHg, p=0.58; Figure 2), its difference with zero in the keratoconus group was statistically significant (paired *t* test, 1.1 ± 0.8 mmHg, p<0.001; Figure 1).

**Table 3:** Comparison of noncorrected and corrected intraocular pressure in patients with keratoconus and age-matched controls

Parameter	Keratoconus		Healthy control		p value^^*^^
	Mean difference (95% CI) (p value)	95% limits of agreement	Mean difference (95% CI) (p value)	95% limits of agreement	
**IOPEhlers-IOPnct**	4.1 ± 2.4 (3.2 to 4.9) (<0.001)^a^	-0.6 to 8.8	0.7 ± 2.6 (-0.2 to 1.6) (0.14)^a^	-4.4 to 5.8	<0.001^b^
**IOPShah-IOPnct**	3.1 ± 1. 7 (2.5 to 3.7) (<0.001)^a^	-0.2 to 6.4	0.7 ± 1.8 (0.1 to 1.4) (0.03)^a^	-2.8 to 4.2	<0.001^b^
**IOPDresden-IOPnct**	2.5 ± 1.4 (2.0 to 3.0) (<0.001)^a^	-0.2 to 5.2	0.6 ± 1.5 (0.1 to 1.1) (0.03)^a^	-2.3 to 3.5	<0.001^b^
**bIOP-IOPnct**	1.1 ± 0.8 (0.8 to 1.4) (<0.001)^a^	-0.5 to 2.7	-0.1 ± 1.1 (-0.5 to 0.3) (0.58)^a^	-2.3 to 2.1	<0.001^b^

*IOPnct=* intraocular pressure not corrected; *bIOP=* biomechanically corrected IOP; *CI=* confidence interval. ^^*^^ Comparison of mean differences between patients with keratoconus and healthy controls.

a Paired *t* test.

b Independent *t* test.

**Figure 1:**
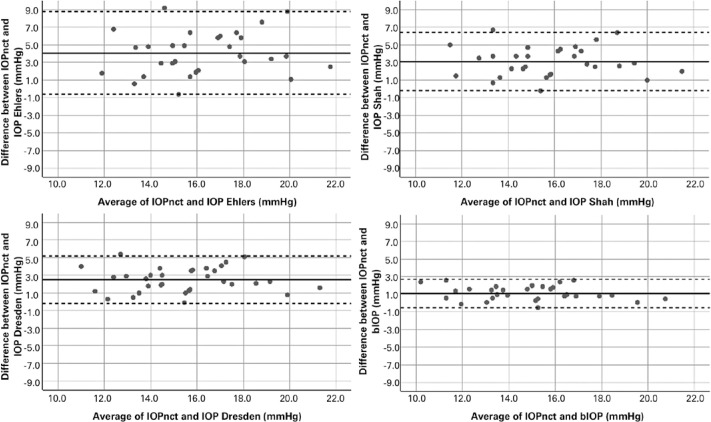
Bland-Altman plots of the agreement between noncorrected intraocular pressure (IOPnct) and corrected IOPs in keratoconus patients (n=34). The *x*-axis represents the average of noncorrected and corrected IOP using different formulas and with measurement using Corvis Scheimpflug Technology. The *y*-axis represents the difference between noncorrected and corrected IOP. The solid line represents the mean difference, and the dotted lines represent the crude 95% limits of agreement.

**Figure 2:**
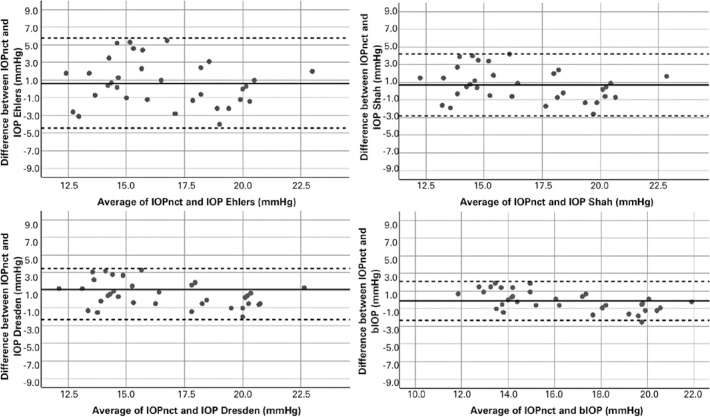
Bland-Altman plots of the agreement between noncorrected intraocular pressure (IOPnct) and corrected IOPs in age-matched controls (n=34). The *x*-axis represents the average of noncorrected and corrected IOP using different formulas and with measurement using Corvis Scheimpflug Technology. The *y*-axis represents the difference between noncorrected and corrected IOP. The solid line represents the mean difference, and the dotted lines represent the crude 95% limits of agreement.

## DISCUSSION

The results of this study showed that some correction formulas, including the Shah^(9)^, Dresden^(10)^, and bIOP formulas^(5)^, eliminated the dependence of IOPnct on corneal thickness and shape in both patients with keratoconus and healthy individuals. However, with all IOP correction formulas, a dependence on corneal biomechanics persisted.

Our results indicating a positive correlation between IOPnct and CCT in both keratoconus and healthy groups^(11-16)^ and a correlation between IOPnct and both anterior and posterior corneal curvature in patients with keratoconus^(6,12)^ are consistent with previous studies. However, no relationship was found with AL or age in the two groups. Indeed, IOPnct strongly correlated with corneal biomechanical parameters in both groups.

One method for reducing the errors of applanation tonometry caused by variability in corneal stiffness features is to use the IOP correction formulas. The results of this study showed that the corrective formulas of Shah^(9)^, Dresden^(10)^, and bIOP^(5)^ corrected the dependence of IOPnct on CCT in both the keratoconus and healthy groups. In addition, the dependence on corneal shape was also corrected in the keratoconus group using all corrective formulas, but none of the formulas corrected the IOP dependence on corneal biomechanical parameters in the keratoconus or healthy groups.

Considering the abnormal biomechanical properties in keratoconus corneas, it seems that comparing IOP readings using different measurement and correction methods with the results of manometry is the only accurate way to determine the most reliable method of IOP correction in keratoconus patients. However, it was not possible to perform manometry in this study; therefore, we adopted another approach to compare the accuracy of the formulas, which could complete the correlation results. In the present study, the healthy group had an IOP range of 11.5-22 mmHg and a mean CCT of 536 µm, which is in agreement with the findings of previous studies^(17,18)^. Based on manometric studies, the measured (noncorrected) and true IOPs are the same in corneas with a central thickness of 520-550 µm^(8,10)^. Accordingly, we assumed that the IOP correction method that produced the highest similarity between IOPnct and corrected IOPs in healthy participants was the most accurate and reliable method of IOP correction. Our results showed that in both the keratoconus and healthy groups, bIOP had the best agreement with IOPnct.

The Ehlers^(8)^, Shah^(9)^, and Dresden^(10)^ formulas are simple linear CCT-based equations that oversimplify the association between IOP and CCT. In some cases, they may overcorrect the IOP for CCT^(19-22)^. We observed this finding in our study, with the presence of a negative correlation coefficient between the IOPs corrected with these formulas and CCT in both the keratoconus and healthy groups. However, this correlation was not statistically significant for the Shah^(9)^ and Dresden^(10)^ formulas. The bIOP formula^(5)^ is the most complex equation used in this study, and it is the only formula in which corneal biomechanics was considered in addition to CCT and age. Using this formula, the association between IOP rea ding and corneal shape and thickness was eliminated; furthermore, in addition to demonstrating good agreement with the IOPnct in the healthy group, its difference with the IOPnct was also nonsignificant. These findings are in agreement with those of previous studies^(5,23)^. In an experimental study of human donor eye globes, Eliasy et al.^(23)^ found that in the range of 10-30 mmHg in healthy eyes, bIOP provided a closer estimation of true IOP as compared with IOPnct and reduced the association with corneal stiffness parameters. A clinical study also showed that the correction algorithm used in the bIOP formula significantly reduced the association between the IOPnct with age and CCT^(5)^.

To the best of our knowledge, this is the first comparison of different IOP correction algorithms in patients with keratoconus. However, some previous studies have been conducted in postrefractive surgery patients, and their results might be comparable with our findings. Refractive surgery techniques make the cornea thin and biomechanically weak. However, in a study of patients undergoing LASIK refractive surgery, Lee et al.^(24)^ found that, as compared with preoperative values, the amount of bIOP remained unchanged after surgery (0.02 ± 1.45 mmHg difference between preand postoperative values), whereas the authors noted a significant difference between preand postoperative values of IOPnct (-2.33 ± 1.54 mmHg).

One limitation of our study is that we did not compare the results of CST tonometry with the results of Goldmann applanation tonometry. However, previous studies have shown high repeatability and reproducibility of CST measurements in both keratoconus and healthy eyes^(25,26)^. In addition, there was no significant difference between Goldmann applanation tonometry and CST measurements, and the results of the two devices were comparable^(27)^. It should also be noted that our study was conducted in subjects with IOPs that were potentially in the normal range. At IOP values greater than 20 mmHg, the amount of correction factor needed for IOP adjustment may significantly increase, and the behavior of the CST noncontact tonometry may change^(28)^. Therefore, the results of this study may be generalized only to the normal range of IOP. Moreover, the sample size in both of our study groups was relatively small, and to confirm our results, further studies with a larger sample size might still be required.

In summary, the results of our study demonstrated that although the measured IOP using CST (IOPnct) was associated with many errors in patients with keratoconus, their corrected forms using CCT-based simple linear formulas did not increase the accuracy of the measurements. In this group of patients, more complex formulas that take into consideration more corneal stiffness parameters in addition to CCT, such as the bIOP formula, might be more reliable and beneficial.
